# D-dimer as a Useful Biomarker in Early Diagnosis of Neonatal Sepsis: A Single-Center Study From Romania

**DOI:** 10.7759/cureus.65213

**Published:** 2024-07-23

**Authors:** Nicoleta Lungu, Daniela-Eugenia Popescu, Florin I Gorun, Georgiana Nan, Zoran L Popa, Aniko Manea, Timea Elisabeta Brandibur, Ana-Maria Cristina Jura, Sergiu Costescu, Biliana Belovan, Marioara Boia

**Affiliations:** 1 Department of Obstetrics-Gynecology and Neonatology, Victor Babeş University of Medicine and Pharmacy, Timisoara, ROU; 2 Department of Obstetrics and Gynecology, Timisoara Clinical Municipal Emergency Hospital, Timisoara, ROU; 3 Department of Obstetrics and Gynecology, Municipal Emergency Clinical Hospital, Timisoara, ROU; 4 Doctoral School, Victor Babeş University of Medicine and Pharmacy, Timisoara, ROU; 5 Department of Obstetrics and Gynecology, Oravita City Hospital, Oravita, ROU

**Keywords:** infections, biomarkers, critical care, neonatology, sepsis

## Abstract

Background: This study evaluates the role of D-dimer in identifying neonatal sepsis and their potential value in clinical decision-making due to challenges in early detection.

Methodology: A case-control study was conducted on 102 neonates at the Children's Clinical Hospital "Louis Turcanu" in Timisoara, Romania, from October 2018 to July 2023. The participants were divided into two groups: those with neonatal sepsis and those without sepsis.

Results: The study found that neonates with sepsis were more likely to be delivered by cesarean section and had higher rates of premature ruptured membranes compared to those without sepsis. The D-dimer biomarker's predictive value for sepsis was assessed using a receiver operating characteristic (ROC) curve, with an area under the curve (AUC) exceeding 0.982 and an optimum cutoff value of 342 ng/mL. An increase in neonatal D-dimer significantly increases the likelihood of sepsis by 2.7% per unit increase. A value above 250 ng/mL indicates a 127-fold increased likelihood of sepsis. The D-dimer's ability to predict mortality in newborns with sepsis is unsatisfactory, with an AUC of 0.528.

Conclusions: D-dimer, a potential biomarker of neonatal sepsis, warrants further clinical investigation to enhance diagnostic sensitivity and specificity, demonstrating its potential in conjunction with other sepsis markers.

## Introduction

Globally, sepsis affects death rates significantly, particularly in neonates, where there are an estimated 3 million afflicted infants and a fatality rate ranging from 11% to 19% [1]. Neonatal sepsis is a medical condition characterized by the presence of bacteria, viruses, or fungi in the bloodstream within the first 28 days of life. Infection is confirmed via the analysis of blood, urine, or cerebrospinal fluid samples [2]. Neonatal sepsis is categorized into early-onset (EOS) and late-onset (LOS) based on presentation time following birth, with LOS occurring after 72 hours, and EOS occurring before 72 hours (although some experts use seven days) [3].

Sepsis has been established as a priority by the World Health Organization (WHO) due to its significant impact on global death rates [4]. The incidence of neonatal sepsis is considered to be influenced by several factors, including birth asphyxia, preterm, low birth weight, delivery sites, method of delivery, prenatal care received, infant mixed feeding, and certain cultural norms for cord care [5-7]. 

According to the third international consensus definition for sepsis and septic shock (Sepsis-3), sepsis is defined as life-threatening organ dysfunction caused by a dysregulated host response to infection [8]. 

Organ dysfunction is defined as a sudden increase in the total Sequential Organ Failure Assessment (SOFA) score by at least 2 points due to an infection [8]. Additionally, septic shock is a subset of sepsis in which the underlying circulatory and cellular/metabolic abnormalities are severe enough to significantly increase mortality [8]. 

Sepsis is a complex host response to an infecting pathogen that endogenous factors may significantly exacerbate [9]. It is known that sepsis causes early activation of both pro- and anti-inflammatory responses. It also causes significant modifications in nonimmunologic pathways like cardiovascular, neuronal, autonomic, hormonal, bioenergetic, metabolic, and coagulation, all of which have implications for prognostic [8]. Sepsis is a syndrome that is shaped by both pathogen and host factors, such as gender, race, age, other medical conditions, and environment [8]. Nevertheless, certain infections may induce local organ dysfunction without eliciting a dysregulated systemic host response [8].

Improved diagnostic criteria and management techniques are required as there are still issues with the early detection, diagnosis, and standardized management of sepsis in children. Diagnosing neonatal sepsis is challenging due to the common occurrence of noninfectious diseases that closely resemble sepsis, particularly in premature newborns, and the lack of effective diagnostic diagnostics [10].

Clinicians in the neonatal setting typically rely on microbiological results rather than organ dysfunction markers for the diagnosis of sepsis, emphasizing the need for better alignment with definitions for adults and children [11].

Recent developments in sepsis recognition tools, including electronic screening tools, have improved the ability to detect and monitor sepsis in pediatric patients [12]. Nevertheless, when contemplating the neonate's clinical presentation, inflammatory markers prove to be valuable in the diagnosis of neonatal sepsis [13]. Inflammatory status was precisely determined by the concentrations of leukocytes, lymphocytes, creatine kinase (CK), C-reactive protein (CRP), lactic acid dehydrogenase (LDH), aspartate aminotransferase (AST), ferritin, and interleukin 6 (IL-6) [14].

D-dimer, a fibrin degradation product, emerges as a promising candidate for fulfilling this role due to its association with the pathophysiological processes underlying sepsis, including coagulation activation, endothelial dysfunction, and fibrinolysis [15]. D-dimer is a biomarker that has received increased attention in recent years due to its potential role in the early detection and predicting outcomes of sepsis [16-19]. While fibrinogen is a crucial protein involved in the clotting process, D-dimer is a fibrin breakdown product that indicates continuous activation of the coagulation cascade and fibrinolysis [20-22].

Although there is increasing evidence to support the use of D-dimer in the diagnosis and prediction of adult sepsis, it is unclear how useful this test is in the neonatal population [23]. Relatively limited research has been conducted on this biomarker's function in neonatal sepsis.

Given the challenges in identifying neonatal sepsis and the potential benefits of early detection, more research is needed into the role of D-dimer in this context. This study aims to evaluate the diagnostic accuracy of this biomarker in identifying infants with sepsis, as well as its potential value in guiding clinical decision-making. Additionally, the predictive value of D-dimer in neonatal mortality associated with sepsis was investigated in this study.

By explaining the D-dimer in the early diagnosis of neonatal sepsis, this research can contribute toward a better prognosis in neonates as well as guide treatment that is supported by scientific evidence.

## Materials and methods

Study design and settings

A single-center case-control study was performed to explore D-dimer as a diagnostic biomarker in neonatal sepsis. This study was conducted on 102 neonates at the Children's Clinical Hospital "Louis Turcanu," Timisoara, Romania, between October 1, 2018, and July 1, 2023. The study was approved by the Ethics Committee for Scientific Research of the Children's Clinical Hospital "Louis Turcanu," Timisoara (No. 6052/March 24, 2022) and by the Ethics Committee of the University of Medicine and Pharmacy “Victor Babes” Timisoara (no. 57/2018)

Participants

Participants were divided into two groups (cases and control) according to the presence of neonatal sepsis, with the case group including 55 consecutive infants diagnosed with proven sepsis (28 with early-onset sepsis [EOS] and 27 with late-onset sepsis [LOS]) and the control group including 47 newborns without sepsis. The newborns in the control group were children admitted to the Children's Clinical Hospital "Louis Turcanu," Timisoara, for the following pathologies: prematurity, neonatal asphyxia, and respiratory distress syndrome. To avoid selection bias, the nearest-neighbor matching method was used to select a control group of newborns.

The criteria for including in the group of cases were as follows: (1) neonates who were admitted to the Children's Clinical Hospital "Louis Turcanu," Timisoara, throughout the research period; (2) the legal representative of the baby had given informed permission; (3) the newborn had a positive diagnostic of sepsis (coded in the electronic patient records using the International Classification of Diseases, 10th Revision (ICD-10) code for newborn sepsis, ICD-10 code: P36.9); (4) the data about laboratory analysis were comprehensive.

Newborns who met the following criteria were included in the control group: (1) admitted during the research period at the Children's Hospital "Louis Țurcanu," Timisoara; (2) informed consent was obtained from the newborn's legal representative; (3) complete data on laboratory tests were available in the electronic database; and (4) had a negative diagnosis of sepsis.

The exclusion criteria for this study were as follows: (1) failure to obtain written permission from the legal representative to use the data, and (2) lack of data.

In patients with elevated D-dimer levels at admission, the presence of disseminated intravascular coagulation (DIC) was considered and excluded in all. DIC was diagnosed when there was evidence of abnormal blood clotting (PT-INR higher than 1.8 or higher than 1.6 in newborns weighing less than 1,500 g), low platelet count (≤70×10^3^/μL or 50% reduction within 24 hours), reduced fibrinogen levels (<50 mg/dL or <100 mg/dL in newborns weighing less than 1,500 g), and increased D-dimer levels (2.5-fold upper limit of normal range). 

After the onset of the COVID-19 pandemic, all participants were tested for SARS-CoV-2 using real-time reverse transcriptase polymerization chain reaction (RT-PCR), with negative results for all participants.

Variables, data sources, and measurement

Using an established data collecting form, two investigators collected data out of neonates’ digital medical records. For every newborn, the following information was obtained: neonatal sex, birth weight, gestational age (GA) at birth, type of birth, APGAR score at 1 minute, data about the mothers (parity, diseases complicating pregnancy, positive cervical cultures, and parity), fetal or pregnancy complications (preterm birth, fetal growth restriction (FGR), premature rupture of membranes, subchorionic hemorrhage) and laboratory data.

At admission, venous blood samples were collected from each infant for laboratory analysis.

The level of D-dimer was determined on admission to the hospital using the ACL TOP 550 (Werfen, Barcelona, Spain). D-dimer samples were collected before the initiation of any treatment.

All sepsis cases were coded in the electronic patient record using the ICD-10 code for neonatal sepsis. The diagnosis of sepsis was established during the hospitalization of patients according to hospital protocol. Initial diagnosis was based on clinical signs (such as temperature instability, lethargy, feeding intolerance, respiratory distress, bradycardia) and laboratory markers (elevated C-reactive protein, abnormal white blood cell count, and hyperbilirubinemia) consistent with sepsis criteria.

The confirmation of sepsis was achieved through the presence of positive blood culture results. The samples for blood cultures were collected before the initiation of antibiotic treatment. In preterm newborns weighing less than 1 kg, a blood culture necessitates the collection of a 1 mL blood sample. For infants ranging between 1.1 and 4 kg, a blood sample volume of 1-2 mL was necessary. The blood sample was introduced into a BacTALERT PF (Organon-Teknika Corp., Durham, NC) blood culture flask and promptly placed on the BacTALERT system (Organon-Teknika Corp.) within a time frame of under two hours from the moment of collection. Blood cultures were performed on all participants included in the study, at the time of hospital admission, before antibiotic treatment.

The variable under investigation was the occurrence of sepsis. The secondary variable of interest was mortality among neonates with sepsis.

Statistical analysis

SPSS 20.0 software (IBM Corp., Armonk, NY) was utilized to conduct statistical analysis. Fisher's exact test was employed to compare categorical variables, which were expressed in both count and percentage formats. The normality of the distribution of continuous variables was determined by the Shapiro-Wilk test, all variables that possess a *P*-value equal to or less than 0.05. Consequently, the continuous variables were expressed as the median and interquartile range (IQR), and the comparison was conducted using the Mann-Whitney test.

The assessment of the predictive performance of D-dimers and fibrinogen for predicting neonatal sepsis was conducted using the area under the curve (AUC) and the receiver operating characteristic (ROC) curve approach. Youden's index was used to calculate the appropriate cutoff values for D-dimers and fibrinogen. Binomial logistic regression was employed to estimate the association. Multivariate logistic regression analyses included confounders variables such as GA at birth, maternal pathology (diabetes mellitus or hypertension), and fetal and neonatal complications (e.g., prematurity, neonatal asphyxia, respiratory distress syndrome, and FGR).

The association between D-dimer values and mortality among neonates with sepsis was assessed using the Kaplan-Meier survival curve and a multivariate Cox proportional hazards model.

## Results

Baseline characteristics

The study included a group of 55 neonates who were diagnosed with sepsis and were monitored throughout their hospitalization. Of the 55 newborns with proven sepsis, 50.9% had EOS and 49.1% had LOS.

Compared to patients in the group without sepsis, newborns with sepsis were statistically significantly more likely to be delivered by cesarean section (*P *< 0.001), and their mothers had a significantly higher rate of premature ruptured membranes (*P *= 0.01) (Table 1). There were no differences between the two groups in terms of gestational age at birth, premature birth rate, APGAR score at one minute, and maternal parity. Furthermore, FGR and subchorionic hemorrhage during pregnancy were significantly more common in the group without sepsis. However, cases of urinary tract infections during pregnancy and positive cervical cultures were present only in the neonatal sepsis group (Table 1). There were no statistically significant differences in baseline characteristics between patients with EOS versus LOS (Table 1).

**Table 1:** Baseline characteristics of 102 neonates included in the study. Continuous variables are expressed as median (interquartile interval). Categorical variables are expressed in absolute count (percentage). *Comparison between the control group and the total sepsis group. **Comparison of the early-onset sepsis group versus the late-onset sepsis group.

Variable	Total (*N* = 102)	Sepsis (*N* = 55)				No sepsis (*N* = 47)	*P*-value*
		Total	EOS (*n *= 28)	LOS (*n *= 27)	*P*-value**		
GA at delivery, median (IQR)	34 (5)	34 (5.5)	34 (6)	35 (6)	0.28	35 (5.5)	0.22
Infant weight (g), median (IQR)	2200 (1007.5)	2300 (1145.0)	2315 (1347.5)	2300 (1120)	0.89	2100.0 (800)	0.65
Male gender, *n* (%)	70 (68.62)	43 (78.18)	22 (78.6)	21 (77.8)	0.99	27 (57.44)	0.18
Cesarean birth, *n* (%)	36 (35.29)	29 (52.72)	14 (50)	15 (55.6)	0.78	7 (14.89)	<0.001
Preterm birth, *n* (%)	84 (17.64)	49 (89.09)	27 (96.4)	22 (81.5)	0.10	35 (74.46)	0.06
Fetal growth restriction, *n* (%)	41 (40.19)	17 (30.90)	7 (25.0)	10 (37)	0.39	24 (51.06)	0.04
APGAR score at 1 minute, median (IQR)	7 (2)	7 (3)	7 (4)	7 (2)	0.20	7 (1)	0.57
D-dimer (ng/mL), median (IQR)	364 (1434.75)	1560 (1987)	1871.5 (2179.0)	1217.0 (2022)	0.30	220 (93)	<0.001
Maternal characteristics							
Parity, median (IQR)	2 (2)	2 (2.5)	2 (3)	2 (2)	0.47	2 (1)	0.34
Pregnancy-induced hypertension, *n* (%)	4 (3.96)	4 (7.40)	1 (3.6)	3 (11.5)	0.34	-	NA
Gestational diabetes, *n* (%)	1 (0.98)	-	-	-	-	1 (2.12)	NA
Premature rupture of membranes, *n* (%)	22 (21.56)	17 (30.90)	11 (39.3)	6 (22.2)	0.24	5 (10.63)	0.01
Subchorionic hemorrhage, *n* (%)	17 (16.83)	5 (9.25)	3 (10.7)	2 (7.4)	0.99	12 (25.53)	0.03
Urinary tract infections, *n* (%)	32 (31.68)	32 (59.25)	15 (53.6)	17 (63)	0.58	-	NA
Positive cervical culture, *n* (%)	8 (7.86)	8 (14.54)	5 (17.9)	3 (11.5)	0.70	-	NA
Blood culture							
Klebsiella, *n* (%)	24 (23.5)	24 (43.6)	13 (46.4)	11 (40.7)	0.11	-	NA
Escherichia coli, *n* (%)	14 (13.7)	14 (25.5)	11 (39.3)	3 (11.1)			NA
Staphylococcus aureus, *n* (%)	1 (1.0)	1 (1.8)	-	1 (3.7)		-	NA
Enterococcus, *n* (%)	3 (2.9)	3 (5.5)	-	3 (11.1)		-	NA
Serratia, *n* (%)	8 (7.8)	8 (14.5)	3 (10.7)	5 (18.5)		-	NA
Enterobacter, *n* (%)	3 (2.9)	3 (5.5)	1 (3.6)	2 (7.4)		-	NA
Pseudomonas, *n* (%)	1 (1.0)	1 (1.8)	-	1 (3.7)		-	NA
Others, *n* (%)	1 (3.7)	1 (1.8)	-	1 (3.7)		-	NA

As for the D-dimer value, it is significantly higher in newborns with sepsis (*P *< 0.001) (Table 1). The D-dimer values in patients with EOS compared to patients with LOS do not show statistically significant differences (Table 1).

In terms of blood culture results, the most common pathogen found was Klebsiella (43.6% of sepsis cases), Escherichia coli (25.5%), and Serratia (14.5%). In terms of comparison between EOS and LOS, Klebsiella and E. coli were more commonly found in EOS patients, while Serratia was more commonly found in LOS patients compared to patients with EOS (Table 1).

The predictive capacity of D-dimer in identifying the likelihood of newborn sepsis

An ROC curve was generated for the D-dimer biomarker to assess its predictive value in determining the occurrence of sepsis. The value of the AUC exceeded 0.982, as can be seen in Table 2. The optimum cutoff value derived from Youden's index was 342 ng/mL.

**Table 2:** Receiver operating characteristics (ROC) curves, prognostic accuracy of D-dimer, and optimal cutoff. AUC, area under the ROC Curve; CI, confidence interval

AUC	95% CI		Cutoff	Youden	*P*-value	Sensitivity	Specificity
	Lower	Upper					
0.982	0.950	1.000	342	0.945	<0.001	0.945	1

A logistic regression was performed to test the discrimination ability of neonatal D-dimer as a prognostic factor of sepsis. The results showed that for every one-unit increase in neonatal D-dimer, the likelihood of sepsis increases by 2.7% (*P *= 0.002) (Table 3).

**Table 3:** The likelihood of neonatal sepsis according to univariate binomial logistic regression. B, log odds ratios (the logistic regression coefficients); SE, standard error; OR, odds ratio; CI, confidence interval

Biomarker	B	SE	*P*-value	OR	95% CI	
					Lower	Upper
D-dimer	0.027	0.009	0.002	1.027	1.010	1.045

Also, multivariate binomial logistic regression, which included gestational age at birth, maternal pathology, and neonatal and fetal pathology (prematurity, neonatal asphyxia, respiratory distress syndrome, and FGR) as confounding factors, showed that each one-unit increase in D-dimer increased the odds of neonatal sepsis by 3% (*P *= 0.005) (Table 4).

**Table 4:** The likelihood of neonatal sepsis according to multivariate binomial logistic regression. Covariates are gestational age (GA) at birth, maternal pathology (diabetes mellitus, hypertension), and fetal and neonatal complications (e.g., prematurity, neonatal asphyxia, respiratory distress syndrome, and fetal growth restriction). B, log odds ratios (the logistic regression coefficients); SE, standard error; OR, odds ratio; CI, confidence interval

Biomarker	B	SE	*P*-value	OR	95% CI	
					Lower	Upper
D-dimer	0.029	0.010	0.005	1.030	1.009	1.051

Further, a logistic regression was performed to determine the effects of the D-dimer value above certain threshold levels on the likelihood of participants developing sepsis.

First, if we consider normal D-dimer values, an increased value above 250 ng/mL (upper limit of the normal value for newborns), the univariate logistic regression model was statistically significant, *χ*^2^ = 60.265, *P* < 0.0005. The model explained 59.6% (Nagelkerke R2) of the variance in sepsis occurrence. Furthermore, the model correctly classified 84.3% of cases.

According to univariate logistic binomial regression, a value greater than 250 ng/mL indicates a 127-fold higher probability of sepsis (Table 5).

**Table 5:** The likelihood of neonatal sepsis according to the standard normal range of D-dimer analyzed using univariate binomial logistic regression. *B*, log odds ratios (the logistic regression coefficients); SE, standard error; OR, odds ratio; CI, confidence interval

Biomarker	B	SE	*P*-value	OR	95% CI	
					Lower	Upper
D-dimer above 250 ng/mL	4.846	1.058	0.000	127.286	15.990	1012.22

Also, the multivariate logistic regression model was statistically significant (χ^2^ = 63.698, *P* < 0.0005). The model explained 62% (Nagelkerke R2) of the variance in sepsis occurrence. Furthermore, the model correctly classified 84.3% of cases.

According to multivariate logistic binomial regression, a value greater than 250 ng/mL indicates a 123-fold higher probability of sepsis (Table 6).

**Table 6:** The likelihood of neonatal sepsis according to the standard normal range of D-dimer analyzed using multivariate binomial logistic regression. Covariates are gestational age (GA) at birth, maternal pathology (diabetes mellitus or hypertension), fetal and neonatal complications (e.g., prematurity, neonatal asphyxia, respiratory distress syndrome, and fetal growth restriction). *B*, log odds ratios (the logistic regression coefficients); SE, standard error; OR, odds ratio; CI, confidence interval

Biomarker	B	SE	*P*-value	OR	95% CI	
					Lower	Upper
D-dimer above 250 ng/mL	4.814	1.080	0.000	123.254	14.830	1024.372

Excellent discriminatory levels are exhibited by both univariate and multivariate logistic regression models that demonstrate the predictive capability of D-dimer above 250 ng/mL for neonatal sepsis; the AUC values exceeded 0.800 (Table 7).

**Table 7:** The receiver operating characteristic (ROC) curves of binomial logistic regression models that utilize D-dimer values exceeding 250 ng/mL to predict neonatal sepsis. AUC, area under the ROC curve; CI, confidence interval; Std. error, standard error

Model	AUC	Std. error	95% CI		*P*-value
			Lower	Upper	
Univariate: D-dimer > 250 ng/mL	0.834	0.044	0.749	0.920	<0.001
Multivariate: D-dimer > 250 ng/mL	0.874	0.037	0.801	0.947	<0.001

Second, if we consider normal D-dimer values, an increased value above 342 ng/mL (value determined by ROC curve analysis), the univariate logistic regression model was statistically significant (*χ*^2^ = 108.469, *P* < 0.0005). The model explained 59.6% (Nagelkerke R2) of the variance in sepsis occurrence. Furthermore, the model correctly classified 96.1% of cases. According to univariate logistic binomial regression, a value greater than 342 ng/mL indicates a 799-fold higher probability of sepsis (Table 8).

**Table 8:** Probability of neonatal sepsis at D-dimer values >342 ng/mL according to univariate binomial logistic regression. *B*, log odds ratios (the logistic regression coefficients); SE, standard error; OR, odds ratio; CI, confidence interval

Biomarker	B	SE	*P*-value	OR	95% CI	
					Lower	Upper
D-dimer above 342 ng/mL	6.683	1.172	0.000	799.0	80.299	7950.3

Also, the multivariate logistic regression model was statistically significant (χ^2^ = 111.570, *P* < 0.0005). The model explained 62% (Nagelkerke R2) of the variance in sepsis occurrence. Furthermore, the model correctly classified 96.1% of cases.

According to multivariate logistic binomial regression, a value greater than 342 ng/mL indicates a 2068-fold higher probability of sepsis (Table 9).

**Table 9:** Probability of neonatal sepsis at d-dimer values >342 ng/ml according to multivariate binomial logistic regression. Covariates are: gestational age (GA) at birth, maternal pathology (diabetes mellitus, hypertension), fetal and neonatal complications (e.g. prematurity, neonatal asphyxia, respiratory distress syndrome, fetal growth restriction); B= log odds ratios (the logistic regression coefficients); SE= standard error; OR= Odds Ratio; CI= Confidence interval

Biomarker	B	SE	P-value	OR	95%CI	
					Lower	Upper
D-dimer above 342 ng/ml	7.635	1.677	0.000	2068.35	77.264	55369.67

Excellent discriminatory levels are exhibited by both univariate and multivariate logistic regression models that demonstrate the predictive capability of D-dimer above 342 ng/mL for neonatal sepsis; their AUC values exceed 0.900 (Table 10).

**Table 10:** The receiver operating characteristic (ROC) curves of binomial logistic regression models that utilize D-dimer values exceeding 342 ng/mL to predict neonatal sepsis. AUC, area under the ROC curve; CI, confidence interval; std. error, standard error

Model	AUC	Std. error	95% CI		*P*-value
			Lower	Upper	
Univariate: D-dimer > 342 ng/mL	0.962	0.022	0.919	1.000	<0.001
Multivariate: D-dimer > 342 ng/mL	0.981	0.011	0.959	1.000	<0.001

The role of D-dimer in predicting death in newborns with sepsis

Among newborns with sepsis, 16 deaths were recorded, with no statistical difference found between the median D-dimer values between the survivors and the deceased.

**Figure 1:**
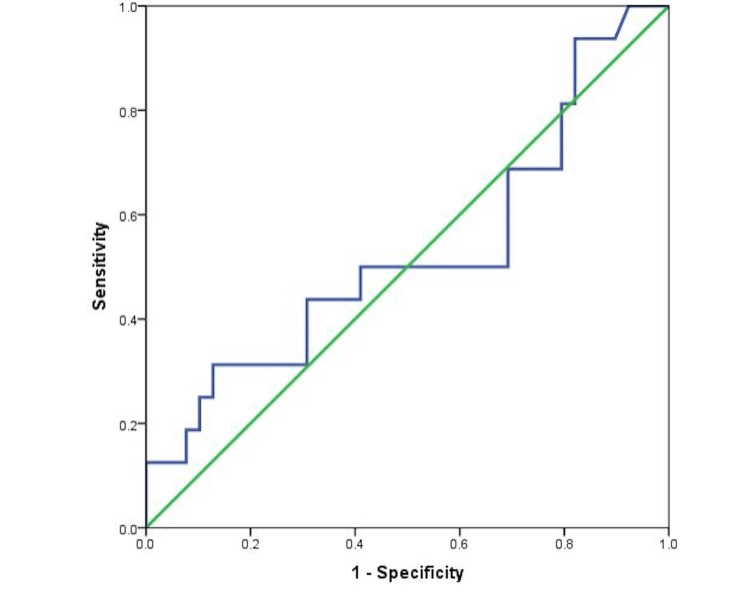
Receiver operating characteristic (ROC) curves of D-dimer in predicting mortality among neonates with sepsis.

Furthermore, the discriminatory power of D-dimer in predicting mortality among the 56 newborns with sepsis was unsatisfactory, with the ROC curve showing an AUC of 0.528 (Figure 1).

Moreover, binomial logistic regression did not show a higher probability of death with increasing D-dimer among septic newborns (odds ratio [OR] = 1.00, *P* = 0.126).

However, ROC curve analysis showed an ideal threshold of D-dimer in predicting mortality among neonates with sepsis of 1,600 ng/mL, showing a sensitivity of 50% and a specificity of 69%.

**Figure 2:**
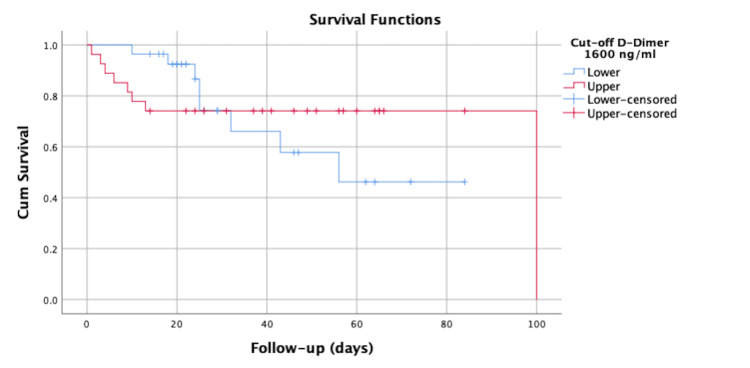
Kaplan-Meier survival curve of neonates with sepsis according to the cutoff value for D-dimer of 1,600 ng/mL.

However, the Kaplan-Meier survival analysis did not show a statistically significant difference between neonates with values below this D-dimer threshold compared to those with values above this threshold (log-rank = 0.102; *P *= 0.75) (Figure 2).

**Figure 3:**
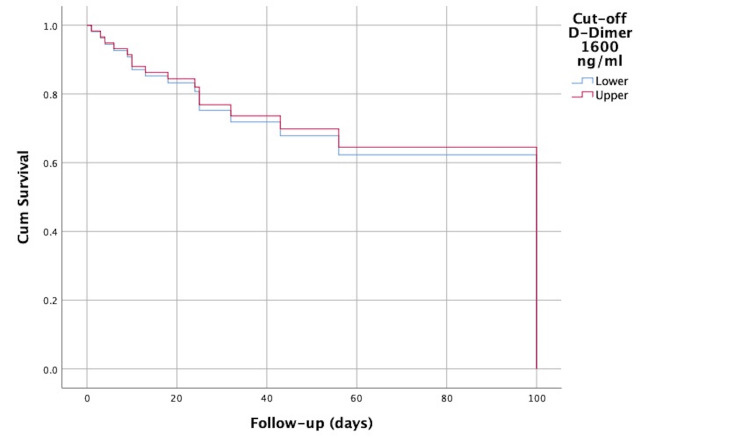
Cox regression model survival curve of neonates with sepsis as a function of the cutoff value for D-dimer of 1,600 ng/mL, adjusted for gestational age at birth.

In addition, multivariate Cox regression analysis, which included gestational age at birth in addition to the D-dimer value, showed that a D-dimer level above 1,600 ng/mL was not an independent predictor of mortality (Figure 3 and Table 11).

**Table 11:** Hazard ratio (HR) of the D-dimer obtained by multivariate Cox regression analysis, adjusted to the gestational age at birth. *B*, log odds ratios (the Cox regression coefficients); SE, standard error; CI, confidence interval

Biomarker	B	SE	*P*-value	HR	95% CI	
					Lower	Upper
D-dimer above 1,600 ng/mL	-0.111	0.536	0.83	0.89	0.313	2.557

## Discussion

This study reported data from 102 newborns, 55 of whom had neonatal sepsis. Baseline data of newborns in our study show that there is a higher rate of cesarean delivery among infants with sepsis. Similarly, Utomo et al. demonstrated that term infants delivered by C-section had a 3.25-fold higher risk of developing EOS in newborns compared to those delivered vaginally [24]. Additionally, in a study conducted by Bejitual et al., cesarean section delivery was identified as a factor substantially associated with neonatal sepsis, with an OR of 2.56 [25]. Nonetheless, while both emergency cesarean section and instrumental vaginal delivery were linked to a twofold increase in the risk of neonatal infection, planned cesarean section was associated with a reduced risk of neonatal infection compared to spontaneous vaginal birth [26].

Furthermore, the findings of our study indicate a correlation between early rupture of membranes and sepsis, with a notably elevated incidence of sepsis observed in neonates delivered from pregnancies that are that involve preterm rupture of membranes. In the same direction, existing literature indicates that pregnant women who experienced prolonged membrane rupture for a minimum of 18 hours before hospital admission, 15 hours during hospitalization, or 48 hours until birth had an increased risk of developing neonatal sepsis [27].

Also in our study, newborns from pregnancies complicated with urinary tract infections and positive maternal cervical cultures were found only in the sepsis group. Transmission of infections from the female genitourinary system to the neonate or fetus is widely recognized as the primary cause of EOS [28]. Common bacterial infections associated with EOS encompass Group B streptococcus (GBS), E. coli, coagulase-negative Staphylococcus, Haemophilus influenzae, and Listeria monocytogenes [28]. Regarding maternal urinary tract infection during pregnancy, Bilgin et al. observed that the presence of maternal urinary tract infection may lead to an increase in urinary tract infection frequency in the newborn [29]. Furthermore, it is worth noting that urinary tract infection frequently leads to sepsis in neonates [30].

In terms of other risk factors, it is well-established that term newborns have a lower risk of sepsis and infection compared to premature neonates [28]. However, the risk of sepsis varies depending on the underlying reason for premature delivery. Infants that were born very preterm due to hypertensive diseases or due to FGR had a higher likelihood of LOS in comparison to infants delivered following preterm labor [31]. Our study, based on baseline analysis, found no correlation between FGR, maternal hypertension, or preterm delivery with the occurrence of sepsis, which contradicts the evidence from existing literature.

Regarding our findings in terms of blood cultures, the most common pathogen found was Klebsiella. Pathogens causing neonatal sepsis vary worldwide. In resource-poor settings, Gram-negative bacteria, particularly K. pneumoniae, are a critical factor in the development of neonatal septicemia, in addition to E. coli and Gram-positive Staphylococcus aureus. Conversely, in developed countries, group B Streptococcus, E. coli, and S. aureus are the primary pathogens responsible for neonatal septicemia [32]. Serratia was another pathogen identified. This agent is a recognized cause of outbreaks in neonatal intensive care units [33]. Also, risk factors for this pathogen are low birth weight and prematurity, or prolonged hospitalization [33]. Most of the subjects included in our study show these characteristics, which may explain these results.

Regarding D-dimer, their value among newborns with sepsis was statistically significantly higher compared to the value among neonates who did not develop septic disease.

Similarly, many other studies state that D-dimer has a notable capacity to accurately forecast the occurrence of sepsis in newborns, exhibiting a high level of sensitivity and a negative predictive value. Therefore, it is imperative to incorporate it into the septic screening protocol for neonates [16,34]. Furthermore, newborns with LOS had significantly elevated levels of D-dimer compared to EOS [16]. In our study, our ROC curve results showed an AUC of 0.982 with a suggested best cutoff point of 342, which gives a sensitivity of 0.945 and a sensitivity of 1. Sensitivity and specificity also remained high (98% and 68%, respectively) when considering the standard cutoff of 250 ng/mL. Other studies in the literature have also shown an increased sensitivity and specificity of 72.7% and 86.7%, respectively, for a cutoff value of 0.75 mg/L [16]. The difference in cutoff values between our study and previous studies may be attributed to variations in patient demographics, illness severity, and testing methods. These disparities emphasize the necessity for more investigation to create standardized thresholds that might be universally applicable across various patient groups and contexts.

Further, in our study, the D-dimer did not show acceptable discriminatory power in predicting mortality among infants with sepsis (AUC = 0.528). Contrary to our findings, another study demonstrated that D-dimer had the highest correlation with in-hospital mortality in critically ill children among coagulation markers [19]. Furthermore, D-dimer is a potentially useful biomarker for severe sepsis in children, according to a study by Abdelaziz et al., which established a mortality cutoff level of 0.87 μg/mL [35]. The divergence between our findings and prior literature may suggest that newborn sepsis is influenced by several factors and involves numerous pathophysiological pathways that contribute to death outcomes. Additional research is necessary to examine the precise elements that contribute to death in infants with sepsis. This will help us better understand the intricate relationship between D-dimer levels and clinical outcomes.

Septic shock and sepsis initiate the activation of various response systems, both locally and systemically, which include fibrinolysis and coagulation. As an indicator of fibrin production and degradation, D-dimer demonstrates the coagulation system's turnover [36]. Thus, suggestions for utilizing D-dimer levels as a method of sepsis monitoring are provided.

The increase in D-dimer levels seen in septic neonates in comparison with the nonseptic neonates underscores the potential of this test to be a dependable biomarker in the diagnosis of sepsis among the neonatal group. Besides helping the decision-making process in treatment, D-dimer has also been useful in giving a better prognosis for patients with neonatal sepsis. By incorporating D-dimer tests into the clinic, healthcare providers will be able to hasten the initiation of appropriate therapy thereby lowering the mortality rate.

There are several limitations to this study. To commence, the research employs a retrospective design, and all data were acquired from a singular clinic. Furthermore, it is possible that the sample size was insufficient to evaluate the prognostic efficacy of D-dimer for mortality in septic neonates, as this cohort comprised only 55 neonates who were diagnosed with sepsis; of these, 16 deaths were accounted for in the analysis. The possible concern was raised by including neonates with prematurity, neonatal asphyxia, and respiratory distress syndrome as part of the control group that these conditions may influence D-dimer levels because neonatal asphyxia has also been linked to coagulation abnormalities [37]. We addressed this issue by using the nearest-neighbor matching method to form the control group, ensuring it was closely matched to the case group based on baseline characteristics. Additionally, we conducted multivariate analyses to adjust for these potential confounders in our statistical analyses. Although these precautions have been taken into account, the impact of these underlying conditions on D-dimer levels cannot be completely ruled out, which is a limitation of the study. Unfortunately, there is a lack of data regarding the classification of cesarean sections as either emergent or non-emergent. This is problematic since noninfectious occurrences that result in emergent cesarean sections could potentially introduce errors when assessing the increase in D-dimer levels.

## Conclusions

As demonstrated in this case-control study, D-dimer was a useful biomarker of sepsis in neonates. Further clinical investigation is warranted to explore the potential of D-dimer in conjunction with other sepsis markers to enhance the diagnostic sensitivity and specificity of neonatal sepsis. A threshold D-dimer value of 342 ng/mL has been shown to be almost perfect in discriminating cases with neonatal sepsis. The standard cutoff value of 250 also showed excellent results in the diagnosis of neonatal sepsis. However, the D-dimer value did not show a predictive value for mortality in neonatal sepsis cases.
